# Clinical Cardiology in South East Asia: Indonesian Lessons from the Present towards Improvement

**DOI:** 10.5334/gh.1133

**Published:** 2022-09-13

**Authors:** Andriany Qanitha, Nurul Qalby, Muzakkir Amir, Cuno S. P. M. Uiterwaal, Jose P. S. Henriques, Bastianus A. J. M. de Mol, Idar Mappangara

**Affiliations:** 1 Department of Cardiology and Vascular Medicine, Faculty of Medicine, Hasanuddin University, Makassar 90245, Indonesia; 2 Department of Cardiothoracic Surgery, Academic Medical Center, University of Amsterdam, 1105 AZ Amsterdam, The Netherlands; 3 Department of Physiology, Faculty of Medicine, Hasanuddin University, Makassar 90245, ID; 4 Doctoral Study Program, Faculty of Medicine, Hasanuddin University, Makassar 90245, Indonesia; 5 Department of Cardiology, University Medical Center Utrecht, 3584 CX Utrecht, The Netherlands; 6 Julius Center for Health Sciences and Primary Care, University Medical Center Utrecht, 3584 CG Utrecht, The Netherlands; 7 Department of Cardiology, Academic Medical Center, University of Amsterdam, 1105 AZ Amsterdam, The Netherlands

**Keywords:** Quality of care, Clinical cardiology practice, South-East Asia, Low- and Middle- income countries, Cardiovascular services

## Abstract

Although cardiovascular care has improved in the last decade in the low- and middle-income countries (LMICs) in South-East Asia Region; these countries, particularly Indonesia, are still encountering a number of challenges in providing standardized healthcare systems. This article aimed to highlight the current state of cardiology practices in primary and secondary care, including the novel cardiovascular risk factors, recommendations for improving the quality of care, and future directions of cardiovascular research in limited settings in South-East Asia. We also provided the most recent evidence by addressing our latest findings on cardiovascular research in Indonesia, a region where infrastructure, human, and financial resources are largely limited. Improving healthcare policies to reduce a nations’ exposure to CVD risk factors, providing affordable and accessible cardiovascular care both at primary and secondary levels, and increasing capacity building for clinical research should be warranted in the LMICs in South-East Asia.

## Background

Cardiovascular disease (CVD) remains a major cause of early death and chronic disability worldwide and was responsible for >17 million premature deaths in 2016 [1,2,3]. Although the CVD mortality rate has substantially declined in high-income countries (HICs) [1,4], it has increased in low- and middle-income countries (LMICs), which include most of the Southeast Asian Countries [5].

The South-East Asia Region (SEAR) has a population of over 600 million people, the majority are younger than 65 years old [6], and the population is rising simultaneously with high poverty levels. In SEAR, the burden of cardiovascular risk factors is growing rapidly [7], CVD manifests at a younger age [8], and premature CVD deaths are increasing at an alarming rate [6,7].

Specifically, life expectancy in Indonesia has risen dramatically since the 1990s – an increase of 8.0 years (95% CI 7.3–8.8) – which has added to the burdens of aging and chronic disease [9]. The top three leading causes of disability-adjusted life-years (DALYs) in Indonesia in 2016 were noncommunicable diseases, including coronary artery disease (CAD), stroke, and diabetes [9].

## CVD risk factors in LMICs

Of the well-established classical risk factors for CVD, hypertension, smoking, and diabetes mellitus are the main risk factors in Asia [10]. A meta-analysis showed that the risk factors for CVD are generally similar in Western and Asian countries, such as hypertension, diabetes, smoking, cardiac causes, and high body mass index [11]. The World Health Organization (WHO) Global Burden of Disease (GBD) 2013 reports that the prevalence of hypertension, hypercholesterolemia, and tobacco smoking has increased in LMICs, while a decreased pattern has been shown in high-income countries [12]. In Indonesia, with over 270 million inhabitants, national health surveys have shown that more than 25% of the general population have hypertension, 30% have high cholesterol and are overweight, 7% have diabetes, 65% of men smoke, and 23% are insufficiently physically active [13]. A recent microsimulation model recommended that reducing complications from diabetes in LMIC need a focus on striving for the initiation and titration of blood pressure and statin medications, rather than focusing on screening for diabetes diagnosis or hyperglycemia control among diabetic people [14].

Given the limited resources, it is generally understood that both primary and secondary prevention of CVD in LMICs are often unaffordable or unavailable [15], and inequality is a major issue [16]. Along with the traditional risk factors, some novel cardiovascular risk factors have recently emerged in LMIC populations. Intrauterine to early childhood factors [17,18], socioeconomic status and dietary mediation [19], psychological distress [20], and air pollution have previously been reported as modifiable determinants associated with the established atherosclerotic CVD in adulthood in LMIC [2]. One study published in 2019 suggests that socioeconomic condition during childhood has a significant inverse association with the occurrence of CVD [21]. Poor nutritional status and higher exposure to infections are believed to be intermediate factors that may induce inflammation in the atherosclerotic process, the underlying pathology in the development of CVD [21,22].

## The common neglected CVD in LMICs

Rheumatic heart disease (RHD) is one of the prominent concerns of CVD in LMICs. Data by Zühlke et al. in the Global Rheumatic Heart Disease Registry (the REMEDY study) showed that the majority of patients in LMICs were of productive age under 30 years old, with a higher proportion of female population. Of these participants, 64% had moderate to severe multivalvular disease, which was complicated by congestive heart failure (33.4%), pulmonary hypertension (28.8%), atrial fibrillation (21.8%), stroke (7.1%), infective endocarditis (4%), and major bleeding (1.9%) [23]. These data also suggested that women with RHD in LMICs largely consisted with childbearing age compared to upper-middle-income countries, which added more complications and burdens linked to this disease to LMICs [23]. In Indonesia, the baseline characteristics were in agreement with the results from the REMEDY study [24]. Recent study by Ambari et al. showed that among RHD patients with mitral stenosis in Indonesia, majority (69.4%) were women, with a mean age of 39.3 – 41.2 years, 18% had hypertension, and 8.1% complicated with DM type 2 [25].

On the other hand, undiagnosed or untreated congenital heart diseases (CHD) are the population in which cardiovascular defects were not screened during pregnancy, nor received treatment during that period. Likewise, CHD has an equivalent impact compared to acquired heart diseases, such as RHD. However, CHD often cannot be prevented. The burden of these patient groups falls profoundly to countries with high fertility rates, which are generally LMICs. Nonetheless, data regarding CHD in LMICs are currently scarce. For this special population in LMICs, cardiovascular care seemingly has limited capability and cannot offer accessible and specific care to those needed.

Database from The COngenital HeARt Disease in Adult and Pulmonary Hypertension (COHARD-PH) registry in Indonesia, illustrated that participants were mostly women with the mean age of 34 years old. Most of the CHD types were secundum atrial septal defect, and at enrolment, these patients had already developed signs of pulmonary hypertension [26,27].

Most CHD cases need surgical treatment. Recent data in 2019 depicted that among 25 CHD patients in Indonesia (mean age 31 ± 15 years), who had either atrial septal defects or ventricular septal defects, none had died during postoperative care in the hospital and the mean hospital length of stay 8.4 ± 3.4 days [28]. However, a longer observational study in Pakistan showed a persistent reduce of health-related quality of life, years after surgical treatment (mean interview time 4.1 ± 1.91 years after surgery) [29].

## Cardiovascular care and practices in LMICs

Currently, there is a wide gap between evidence-based recommendations and clinical practice in most less-affluent countries. Treatment of major CVD risk factors remains suboptimal, and only a few patients who are treated achieve the target levels for blood pressure, glucose, and cholesterol [30]. On the other hand, overtreatment can occur with the use of non-evidence-based protocols.

**Image 1:**
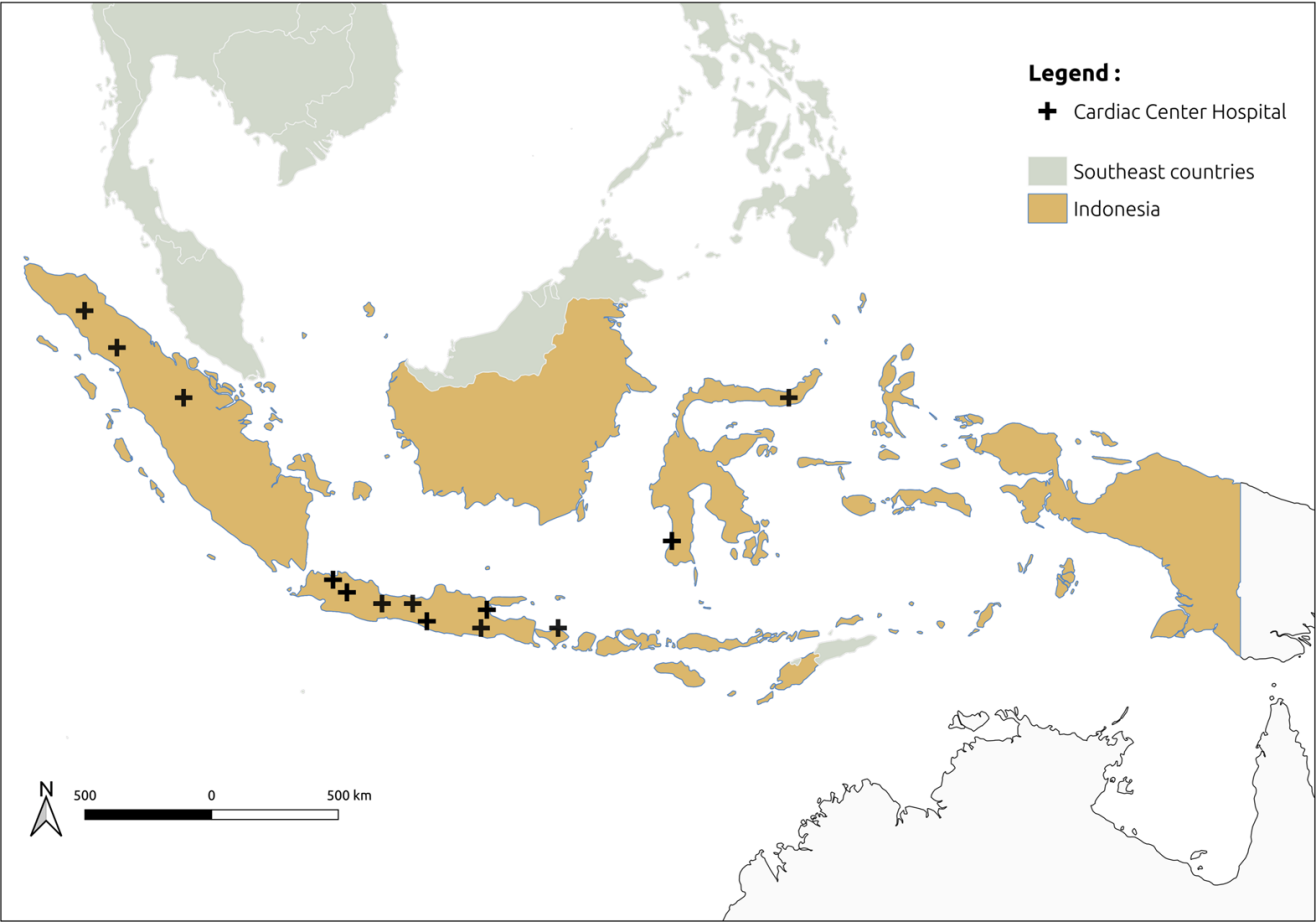
Indonesia in South-East Asia Region (SEAR), with cardiac centers across the archipelago.

Previous research have shown that there is an insufficient access to the guideline-recommended treatment for combating CVD in LMICs [31,32]. In these countries, the healthcare infrastructure is weak, the number of cardiologists is low, and access to quality and timely medical care is still a major challenge. For example, in Indonesia, in 18 provinces, 94.1% of households are located >5 km from any primary care center or hospital with only very minimal means of transportation [33]. A picture of how big Indonesia as an archipelago country with existed cardiac centers across the nation is illustrated in Image 1.

A previous study reported that almost 70% of the study population in Indonesia who were at moderate-to-high risk for CVD failed to access cardiovascular care [34]. Higher income, possession of health insurance, and residence in urban areas are significant predictors of the fulfilled needs of cardiovascular services in Indonesia [34]. The inequality of cardiovascular care at the primary and secondary levels, particularly in rural and remote areas, is vastly insufficient to address the increased rate of CVD in this country. Consequently, most of these patients are concentrated in urban secondary care or tertiary hospitals, resulting in long waiting lists [34].

In the majority of LMICs, primary care capacity is lacking, and the health outcomes are poor [35]. Primary and secondary care tends to be delivered by a mix of public and private sector health care providers. To date, the main focus of CVD care in most LMICs is curative and hospital-centered. Therefore, the majority of people who are at high risk for CVD remain unrecognized, while those with established CVD or chronic risk factors have less education or limited access to essential therapy [4].

In Indonesia, patients with better financial support tend to have CVD treatment abroad in high-income countries, such as Singapore and Malaysia. The main reasons are the long waiting lists due to undersupply of specialized cardiovascular facilities and the assumption that the medications, high-tech interventions, and services available abroad are superior to those in their home country [33].

In Indonesia, several schemes for funding the healthcare had been implemented since 1947. However, those health insurances only covered specific groups of population, for example government and military personnel [36]. Starting in January 2014, the Indonesian government launched the National Social Health Insurance (known as the *Jaminan Kesehatan Nasional*), targeting the poor and near-poor. Since the inception of this program, total enrollment has increased from 86.4 million people in 2014 to 111.6 million in November 2017 [37]. However, issues with coordination between levels of government and inadequate staffing have led to the inequality in access and availability across Indonesia.

Health insurance in South-East Asian LMICs is regulated differently than equivalent insurance in the high-income world. In LMICs, healthcare coverage is still incomplete. Insurance companies tend to spend substantial amounts of money financing curative and rehabilitative treatments, rather than health promotion and preventive strategies.

In most LMICs, patients are diagnosed at a late stage of their CVD and often present to hospitals with acute events or long-term complications [4]. Based on our previous study, most CVD patients in Indonesia present at the hospital at a relatively young age, predominantly with unstable CAD and severe illness. There is generally a significant time delay from disease onset to hospital admission, and they have rarely received treatment recommended by the guidelines [33]. As a consequence, if CVD is at an advanced stage, expensive high-technology interventions may be necessary, e.g. coronary artery bypass graft (CABG) [4]. This condition makes the ‘out-of-pocket’ expenditure even higher, especially for those without health insurance.

A recent review has reported that to date, cardiac surgery is unreachable for 93% (>6 billion people) of the population in LMICs when needed [38,39]. Doubt regarding the need, feasibility, and high costs associated with cardiac surgery in LMICs exists [38]. However, despite the more invasive nature of CABG compared to percutaneous coronary intervention (PCI), CABG can be better option than PCI in LMICs due to its outcomes and cost-effectiveness [40]. Furthermore, thrombolysis which administered during preparation for PCI can have similar outcomes as primary PCI and thrombolysis followed by PCI [41,42]. When multivessel CVD is found during PCI, CABG can be performed as a further treatment option [43].

The occurrence of acute CVD, such as acute coronary syndrome (ACS), is unpredictable and is often fatal. Good outcomes depend on timely management, adequate and accessible facilities, a well-established transportation system, 24-hour services from professional providers, and patient awareness of the symptoms of CVD [44]. Unfortunately, the service system for acute CVD, especially ACS, in Indonesia is still left behind. Generally, the utilization of cath lab is suboptimal, the 24/7 system is not applied, and fast response of the healthcare providers for undergoing primary or rescue PCI remains abandoned. In most LMICs in SEAR, particularly in Indonesia, the 24/7 system for rescue or primary PCI can only meet in tertiary or national level of cardiac center [45].

In contrast, during a chronic phase of CVD, such as congestive heart failure (CHF), stable CAD, and stroke, screening for risk factors, systematic monitoring for complications, and engaged patient self-care and adherence to after-discharge medications are required. Our latest findings have shown that non-adherence to after-discharge medications is a strong predictor for medium-term mortality in patients with CAD in a poor South-East Asian setting [46]. Meanwhile, a recent systematic review has suggested that the use of evidence-based medications for heart failure across LMICs tends to be suboptimal [44].

The WHO has reported that current LMIC health systems rarely meet the standard requirements for chronic care [4]. Data from the Prospective Urban and Rural Epidemiology (PURE) study indicate significant gaps in the study population: there were 50–75% of people with existing CVD received none of the recommended medicines for secondary prevention [15,21,47]. These studies also highlight that the use of CVD medications in women is lower than in men [1,47]. Patients with CVD are often prescribed several long-term medications for primary or secondary prevention, making adherence to medications a key challenge in reducing the CVD burden in LMICs [46].

Once known as a disease of the rich, mounting evidence has indicated that the poor are now at a greater risk for CVD. The inverse relationship between socioeconomic status and CVD incidence and mortality, has been shown across several populations [12]. Smoking, hypertension, dyslipidemia, heavy drinking, obesity, diabetes, and inflammatory markers are more prevalent among the poor, not only due to excessive exposure, but also due to lack of physical activity and less opportunity to obtain healthier foods and access to preventive care [12]. Regardless of location, the urban poor in LMICs also have higher rates of CVD [12].

Generally, in LMICs, most people have a lower educational and socioeconomic status compared to those from high-income countries. Patients in LMICs are rarely aware of the long-term consequences of cardiovascular risk factors, nor do they understand the importance of primary and secondary prevention. In addition to educational and financial level, the opinions of family and relatives, religious beliefs, social values, local culture, and fear of the hospital and medical interventions have always been influential factors, meaning that patients often end up with reluctant or rejection to recommended treatment. In our previous study, participants with lower educational attainment and lower-income was the majority of ACS patients who chose not to undergo CAG and subsequent intervention [48]. Inversely, a cross-sectional study by Peiris et al. in 2020, showed that higher educational level and being on employment associated with a lower risk of CVDs in most LMICs [49].

## Secondary cardiac care

There has been an increasing focus on secondary cardiac care in LMICs in recent decades. Improvements in infrastructures and healthcare policies have been implemented in SEAR. However, even though cardiovascular care has improved rapidly in LMICs, it is still lagging behind the high-income countries.

**Image 2:**
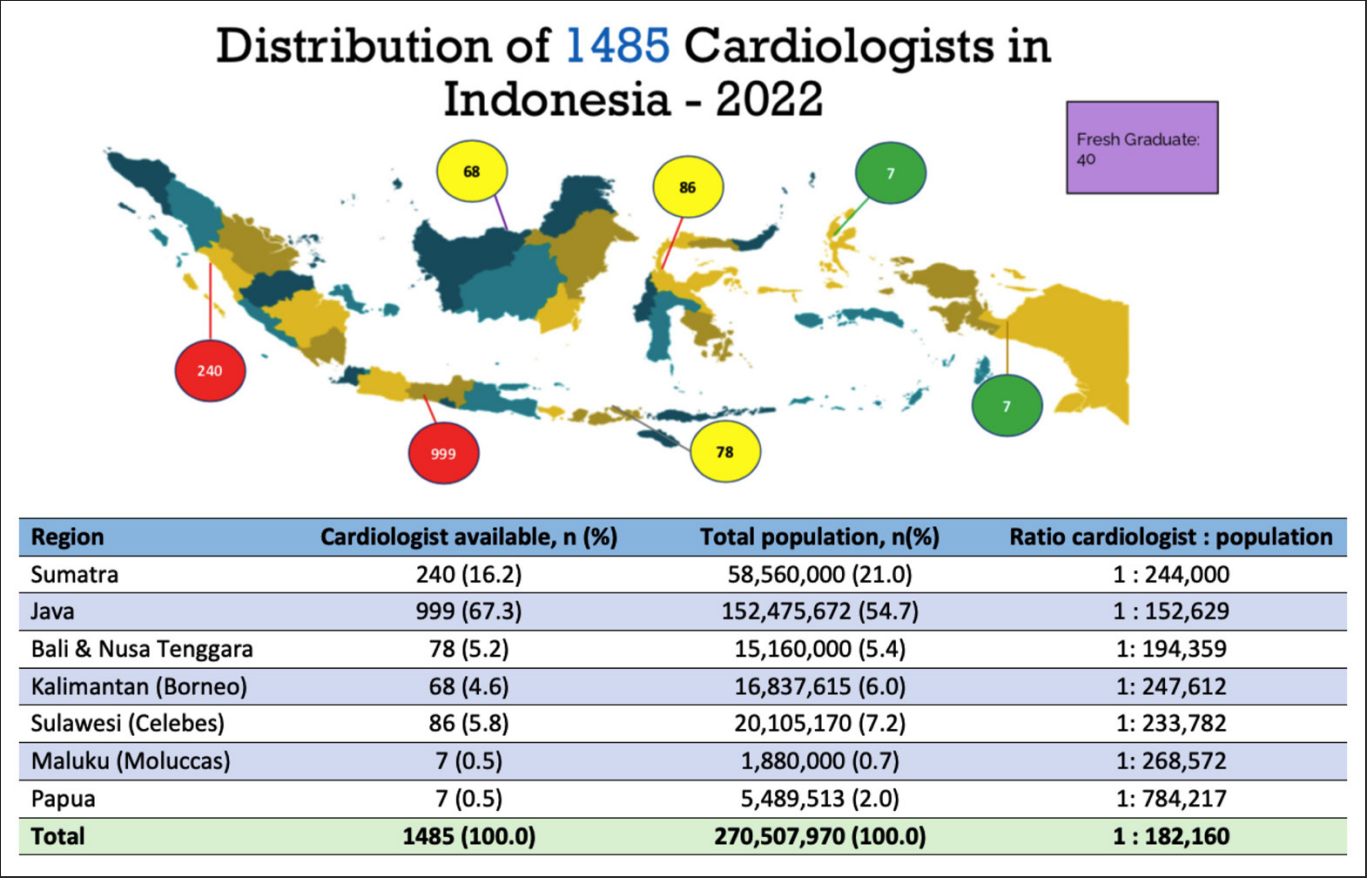
Distribution of cardiologists in Indonesia.

In Indonesia, the number of cardiologists is still lacking with inequal distribution between regions. Similar with other LMICs, the cardiologists predominantly work in big cities with sufficient infrastructure. In Indonesia, approximately 85% of the cardiologists live in West Indonesia, with >65% residing in Java. Inequality of the distribution of cardiologists in Indonesia is presented in Image 2. By increasing the number of cardiologists, the ratio between CVD patients and expert doctors can be further reduced, and therefore increased the quality of care and lowered the burden on the existed cardiologists.

The standard national guidelines for cardiovascular care are commonly found in South-East Asian countries, and the majority are adopted from the Western world, with adjustments for available infrastructures and local settings. Unfortunately, in most LMICs in SEAR, compliance to the guidelines and protocols is still low. The authority of medical doctors is still considered to be the main factor in disease management and decision-making.

Taking into account the aforementioned aspects, although secondary and tertiary cardiovascular care has vastly improved in South-East Asian LMICs over the past decades, these countries are still encountering a number of challenges in providing standardized and appropriate healthcare systems [45]. The key problems of cardiovascular care practices in LMICs are presented in Table 1.

**Table 1:** Key problems of cardiovascular care in Indonesia, based on our observation.


HEALTHCARE SYSTEM

- Low access to care - Healthcare facilities: inequality and distance (problems with transportation) - Unavailable or unaffordable CVD services in primary care - Lack of collaboration between hospitals and primary care doctors - Utilization of ambulance is underused, especially in rural areas - Immature health insurance coverage, or unaffordable health insurance - Lack of surveillance and disease monitoring in the population - Primary care has insufficient capacity to diagnose, monitor, and manage CVD burden, including hypertension and diabetes

**HEALTHCARE PROVIDERS**

- Limited availability of health personnel, especially in remote areas - Lack of standardization among healthcare providers and experts in cardiology practice - Authority in decision-making, ignoring the guideline standards - Poor management of after-discharge care

**PATIENTS**

- Low awareness of CVD symptoms and risk factors - Financial problems: high out-of-pocket expenditure or expensive cost of essential treatments, such as medicines for hypertension, diabetes, and cholesterol - Low adherence to medications for primary and secondary prevention - Low level of education of the patients and family, in the context of adherence to guideline recommendations


## Recommendations for improving cardiovascular care in LMICs

Challenged with low healthcare budgets and poor infrastructure, it is far more difficult to improve the quality of care in LMICs. A sensible attempt to answer this challenge is to implement the most applicable and effective interventions adopted from the Western world, rather than recommending unproven interventions [44]. The program should not be expensive and should be easily applicable with respect to the limitations of the available resources. Although adopting clinical practice guidelines from high-income countries appears to be a great opportunity, the adoption of guidelines and interventions should be adjusted to the local settings and needs [44]. Specifically, in this context, we have proposed a framework to improve the quality of cardiovascular care in Indonesia based on the local evidence acquired during our research (see Table 2).

**Table 2:** Proposed conceptual framework for improving the quality of cardiovascular care in Indonesia (based on the local needs and settings).


HEALTHCARE SYSTEM

- Reduce delay in hospital admission, especially for patients with acute CVD - Reduce administrative and insurance barriers in the hospital - Accessible and affordable cardiovascular care at Puskesmas (primary care centers), such as ambulatory ECG, standardized laboratory checks - Improve access to revascularization services - Implementing telemedicine or mobile-health program through SMS/phone calls to improve lifestyle and adherence to after-discharge medications - Tele-ECG monitoring and consulting at the primary care level - Reliable patient registries should be available in a computerized format - Improve data collection for healthcare utilization (i.e. population surveillance, CVD registry, death registry, etc) - Preventive strategies: optimizing health and nutrition in pregnant women (including vaccination prior to or during pregnancy and adequate treatment for maternal high blood pressure) ▪ Lifestyle improvement: reduce consumption of fatty or deep-fried food, sugar, salty or MSG-contained, and fast food; promote active lifestyle; smoking cessation ▪ Adequate treatment for hypertension and diabetes: accessible care and diagnostic tools and essential medicines at primary level

**HEALTHCARE PROVIDERS**

- Timely and standardized initial management for acute CVD - Implement clinical practice guidelines and improve adherence to the guideline recommendations - Improve hospital discharge planning and transition to chronic care - Update knowledge and skills

**PATIENTS**

- Improve awareness of acute CVD symptoms - Improve home monitoring and awareness of CVD risk factors - Optimizing patients’ adherence and engagement with long-term medications - Improve lifestyle


Currently, the question of whether to target populations on a wide scale or focus on high-risk and diagnosed individuals to reduce the CVD burden is still a matter of debate in resource-poor settings. Individual-scale programs are appropriate for short-term purposes, while population-wide strategies are appropriate for long-term investment. Learning from the high-income world, a relatively new approach that may well be most effective is to combine population- and individual-level strategies [12]. In high-risk populations, blood pressure-lowering intervention was the most cost-effective option [50]. However, simply replicating the prevention programs from high-income countries could become another challenge and is not realistic considering resource discrepancies.

Considering the competing priorities (i.e. maternal and child health problems and infectious diseases) in Indonesia, we suggest that optimizing maternal health and nutrition during pregnancy may reduce or delay the development of CVD in offspring [17]. Our previous study also confirms that higher levels of exposure to infection during childhood and adolescence are associated with the occurrence of early coronary heart disease in adult life and may potentiate the effects of traditional CVD risk factors [18]. Vaccination and adequate treatment for those who are vulnerable – pregnant women and young children with infectious disease – are of practical importance, especially in areas where the prevalence of infections is high [17]. In the context of CVD prevention, the prevention of outbreaks of infectious disease today could be a valuable long-term investment in the future.

With regards to RHD, prevention is crucial and routine screening in the excessive-cases area is recommended. Since rheumatic fever is mostly experienced by school-aged children, screening in school to discover those in need of secondary prophylaxis might be the most appropriate within a limited resource country. Along with it, training specialization on echocardiography for diagnostic of RHD amongst cardiologists, general physicians, and nurses could probably help reach the rural area [51]. Currently, Jones criteria for rheumatic fever diagnosis are less sufficient to diagnose recurrent rheumatic fever [24], thus adapted clinical pathway according to data from local studies needs to be developed. Aside from that, ensuring the availability of medicines, both for treatment and secondary prophylaxis are also priorities. A campaign to recognize the symptoms and signs of rheumatic fever or RHD, provide a proper treatment, and accurate diagnosis of new or recurrent cases are also relevance to be emphasized at the first-line healthcare level.

For congenital heart disease (CHD), besides enhancing surgical access and infrastructure for CHD treatment, promotion by encouraging young women to prepare well for a pregnancy, incorporating healthy lifestyle and avoiding the use of certain medications during pregnancy is recommended. Furthermore, access to routine check-ups, 20-weeks organ screening, and young babies’ routine examination by pediatrists are also encouraged. A previous study also suggested the possibility of screening for CHD in elementary school children by means of cardiac auscultation and 12-lead electrocardiography [52].

The availability and access to echocardiography to detect RHD, congenital defects, and valvular heart disease in rural areas in Indonesia is quiet promising. Lots of patients from remote areas are referred to tertiary cardiac center for advanced diagnostics and treatments with the use of echocardiography.

A recent systematic review has shown that pharmacological interventions targeting blood pressure, lowering cholesterol, and anti-platelet aggregates were predominantly used in the context of economic evaluation for primary and secondary prevention in LMICs [50]. Upcoming approaches should consider the nonpharmacological (i.e. behavioral and lifestyle) interventions, for example, smoking cessation, improvement of physical activity, dietary practices, and weight control. In Indonesia, recent evidence has shown that dietary risks were a leading contributor to the DALY burden, accounting for 14% of total DALYs in 2016 [9].

As the frontrunner for delivering healthcare services, primary care should be more active and involved in the execution of preventive strategies in LMICs. In Indonesia, a country with the largest archipelago in the world [9], distance and infrastructures are the major issues where accessing care is concerned. In 2018, there were 9,993 primary care centers (known as Puskesmas), of which 3,623 (36%) were inpatient centers scattered unequally throughout Indonesia [53]. The quantity and quality of the primary care facilities in Indonesia should be improved, particularly in remote areas.

**Image 3:**
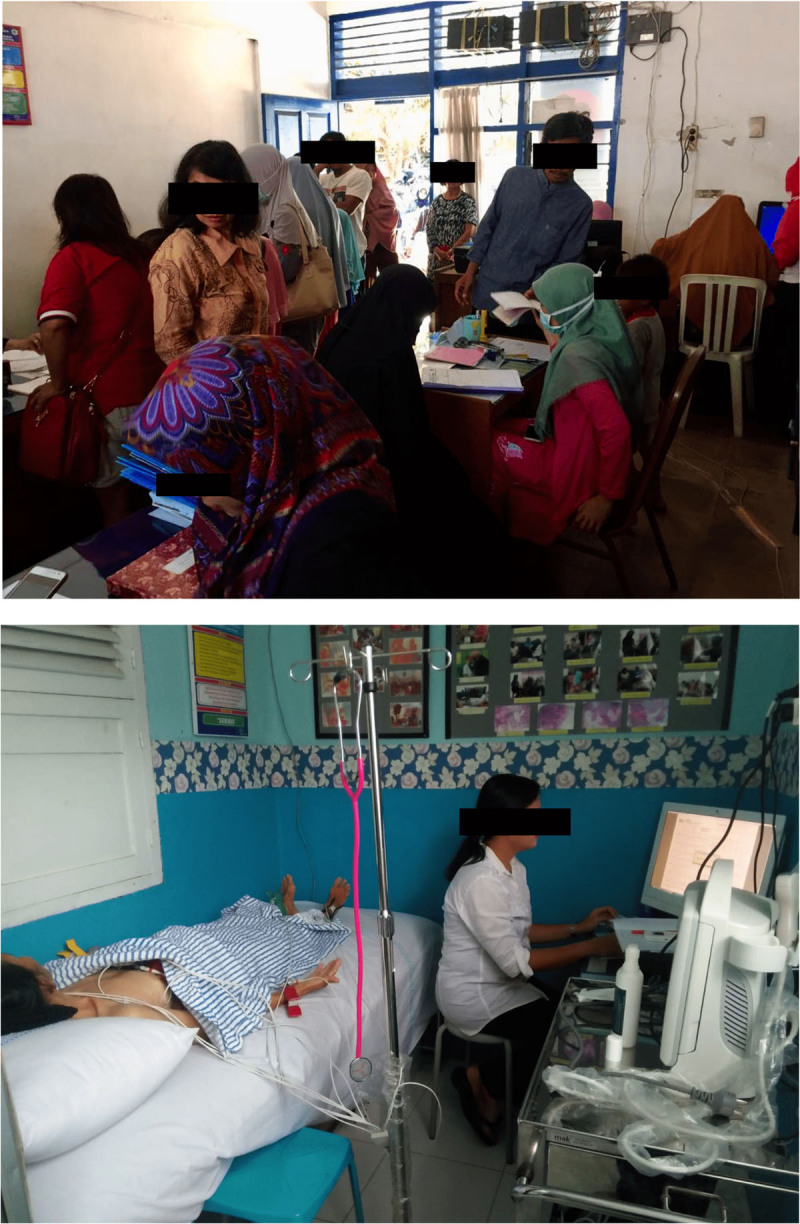
Routine healthcare services and implementation of tele-ECG program in a primary care center (Puskesmas) in Indonesia.

Telemedicine and e-health could deliver both standards and specialized care nearer to the patients. Implementing telemedicine, such as tele-electrocardiography (ECG) at the primary care level could be a cost-effective strategy to detect CVD in high-risk patients and prevent overtreatment after a specialized cardiology consultation. The Makassar Telemedicine Program is our first experience in implementing tele-ECG consultations in East Indonesia. Our findings suggest that despite a shortage of cardiologists and limited resources, tele-ECG is helpful to support general practitioners at primary care centers to perform prehospital triage and refer the indicated patients to secondary or tertiary care hospitals for further specialized treatment [54]. Image 3 shows the routine healthcare services and implementation of tele-ECG program at primary care center in Indonesia.

The ultimate responsibility for better nationwide health systems depends on governmental efforts, which should involve all sectors of society. In 2017, the Indonesian government launched Presidential Decree No. 1 to encourage a healthy lifestyle. This focused on physical activity, a healthy diet, and early detection of health problems [9]. Further legislation supporting the reduction of cardiovascular risk factor burden is also warranted.

## Future directions

In terms of future research, building the research capacity of local staff in the cardiovascular field by means of training courses, seminars, and workshops will empower local researchers to generate local evidence. Capacity building is crucial for the independency and sustainability in conducting clinical research in LMICs. Countries should consider further investment in CVD surveillance and population-based registries to benchmark their efforts in reducing CVD mortality and morbidity.

**Image 4:**
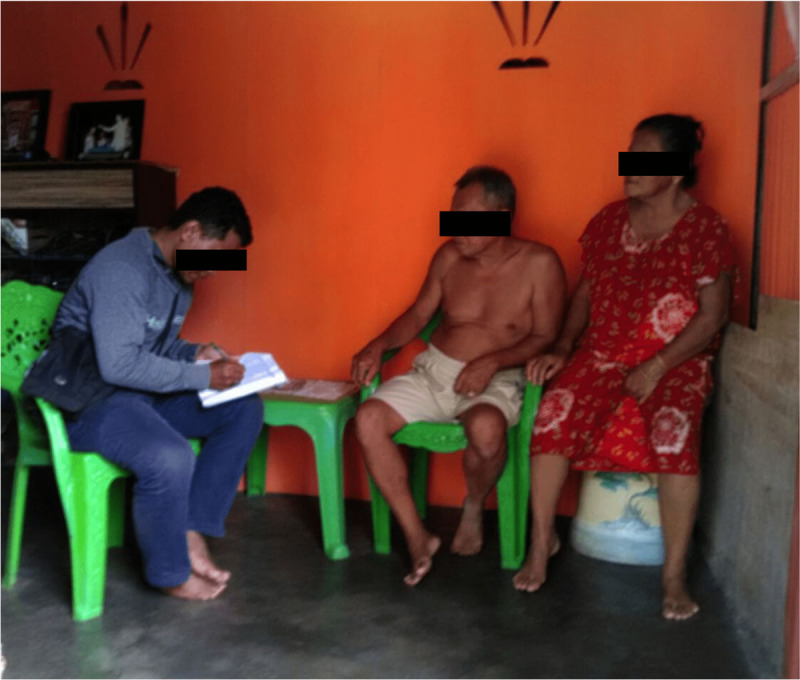
Primary care nurse performing patient follow-up (for cohort research) through home visit.

Based on our experience, conducting clinical research in a resource-limited setting is somehow rather ‘exhausting’. Local researchers are forced to start everything from the scratch. Unavailable standard systems to perform patient follow-up in hospitals and primary care centers required the researchers to be more active in reaching patients or their family members to obtain follow-up data. Image 4 presents the primary care nurse performing patient follow-up through home visits.

An interesting publication in The Lancet has highlighted that as the second most populous LMIC after India, Indonesian voice is too quiet in the global conversation of health and medical science [55]. Research culture and allocation for health research should be encouraged [55]. Increasing the number of health and clinical research modules in university curricula, good mentorship, knowledge-sharing events, and access to free knowledge resources are several strategies to improve the research culture [56]. Despite all the steps, personal motivation and research awareness is also essential.

Clinicians and practitioners in LMICs often undervalue the benefits of evidence-based medicine, and it is probably erroneously perceived that guidelines could limit their autonomy in treating the patients [56]. Most of them regard the research as an academic pastime only, rather difficult, and always time-consuming. Naturally, an extremely heavy workload and other duties in hospitals limit the opportunity and willingness to undertake research. Research is often regarded as a side job that is needed to compete with private practice and other priorities [56]. The lack of resources and recognition for conducting research in the public hospital system increase reluctance for academicians and clinicians to conduct the clinical research. In other systems, clinical research is considered a tool to measure and communicate the quality of care. We have summarized the key challenges of conducting clinical research in low-to-middle-income settings based on our own experience in Table 3.

**Table 3:** Key challenges of conducting clinical/hospital-based research in low-to-middle-income settings in Indonesia (learning from our experience).


SYSTEM/ENVIRONMENT

- Lack or unavailable patient registries/computerized database - Limited and unreliable paper-based medical records - Limited or unavailable population surveillance - Unavailable/limited death registry - Inadequate research infrastructures: research devices/tools should be shared with routine services in the hospital - Less support from the hospital environment (e.g. administrative barriers) - Lack of supportive facilities: poor internet connection, limited access to knowledge resources (e.g. international journals)

**RESEARCHERS (ACADEMICIAN AND CLINICIAN)**

- Limited dedicated time for research, particularly if the investigators are clinicians - Lack of peer supports - High-cost expenditures (e.g. hiring research assistant, laboratory expenses, rewards to patients/participants, high publication costs) - Research community is less familiar with the scientific language of English - Low ‘research and writing’ culture

**PATIENTS**

- Negative attitude towards research: low participation rate, patients/family members’ mistrust, and negative prejudice, rejection for follow-up - High rate of lost-to-follow-up, in particular for those from rural areas - Informed consent issues: difficulty in getting approval from patients and family members (especially if intervention is needed), verbal informed consent for illiterates - Low education and social values are strong influencers (more comprehensive communications are needed for illiterates/low-educated participants) - Language barriers: some patients/participants only use their local/traditional language, not Bahasa Indonesia


Institutions should provide a supportive environment, allocate funding for research, and link research to career enhancement. International support and collaboration are essential for capacity building and co-shaping infrastructure in many ways. For international partners, bilateral academic partnerships could provide the capacity-building platform, for example, the student exchange program, sending residents from LMICs to join didactic courses and hands-on clinical experience in HICs and conducting joint research collaboration to answer the common CVD problems in LMICs. With the aforementioned examples of local evidence, we intend to show that international collaborative projects and local initiatives in the field of medical research are beneficial to Indonesian patients and clinical science in general.

## Conclusions

There is a wide gap between evidence-based recommendations and clinical practice in most of LMICs in South-East Asia, particularly Indonesia. Given the limited resources, both primary and secondary prevention of CVD in LMICs are often unaffordable or unavailable. Access to the guideline-recommended treatment for combating CVD in these developing LMICs is insufficient. In these countries, the healthcare infrastructure is weak, the number of cardiologists is low, and access to quality and timely medical care is still a big challenge. Exploring the local evidence through capacity building and clinical research, implementation of the standard guidelines with adaptation to available resources and local settings, and involvement of the government and stakeholders are needed to reduce the CVD burden in these resource-poor South-East Asian countries.

## Figures and Tables

**Table:** 

LMICs	: Low- and middle-income countries
CVD	: Cardiovascular disease
WHO	: World Health Organization
CAD	: Coronary Artery Disease
HICs	: High-income countries
SEAR	: South-East Asia Region
CI	: Confidence Interval
DALYs	: Disability-adjusted life-years
GBD	: Global Burden of Disease
RHD	: Rheumatic Heart Disease
REMEDY	: Global Rheumatic Heart Disease Registry
CHDs	: Congenital Heart Defects
COHARD-PH	: COngenital HeARt Disease in Adult and Pulmonary Hypertension
NCDs	: Noncommunicable diseases
CABG	: Coronary Artery bypass Graft
PCI	: Percutaneous Coronary Intervention
CHF	: Congestive Heart Failure
PURE	: Prospective Urban and Rural Epidemiology
MONICA	: MONItoring of Trends and Determinants of CArdiovascular Disease
ECG	: Electrocardiography
GPs	: General Practitioners
